# Direct Enantiomeric Separation and Determination of Hexythiazox Enantiomers in Environment and Vegetable by Reverse-Phase High-Performance Liquid Chromatography

**DOI:** 10.3390/ijerph17103453

**Published:** 2020-05-15

**Authors:** Ping Zhang, Sheng Wang, Dongmei Shi, Yangyang Xu, Furong Yang, Xile Deng, Yuhan He, Lin He

**Affiliations:** 1 Key Laboratory of Entomology and Pest Control Engineering, College of Plant Protection, Southwest University, Chongqing 400715, China; zpcauz@163.com (S.W.); shidm48@163.com (D.S.); zp8708@163.com (Y.X.); yfr200111@163.com (F.Y.); hm20161027@163.com (Y.H.); 2 Academy of Agricultural Sciences, Southwest University, Chongqing 400715, China; 3 State Cultivation Base of Crop Stress Biology for Southern Mountainous Land of Southwest University, Southwest University, Chongqing 400715, China; 4 Key Laboratory for Biology and Control of Weeds, Biotechnology Research Institute, Hunan Academy of Agricultural Sciences, Changsha 410125, China; dengxile@hunaas.cn

**Keywords:** hexythiazox, enantiomeric separation, residue analysis, vegetable, environment

## Abstract

In the present study, the direct enantiomeric separation of hexythiazox enantiomers on Lux cellulose-1, Lux cellulose-2, Lux cellulose-3, Lux cellulose-4, Lux amylose-1 and Chirapak IC chiral columns were carefully investigated by reverse-phase high-performance liquid chromatography (RP-HPLC). Acetonitrile/water and methanol/water were used as mobile phase at a flow rate of 0.8 mL·min^−1^. The effects of chiral stationary phase, temperature, thermodynamic parameters, mobile phase component and mobile phase ratio on hexythiazox enantiomers separation were fully evaluated. Hexythiazox enantiomers received a baseline separation on the Lux cellulose-3 column with a maximum resolution of R_s_ = 2.09 (methanol/water) and R_s_ = 2.74 (acetonitrile/water), respectively. Partial separations were achieved on other five chiral columns. Furthermore, Lux amylose-1 and Chirapak IC had no separation ability for hexythiazox enantiomers when methanol/water was used as mobile phase. Temperature study indicated that the capacity factor (k) and resolution factor (R_s_) decreased with column temperature increasing from 10 °C to 40 °C. The enthalpy (ΔH) and entropy (ΔS) involved in hexythiazox separation were also calculated and demonstrated the lower temperature contributed to better separation resolution. Moreover, the residue analytical method for hexythiazox enantiomers in the environment (soil and water) and vegetable (cucumber, cabbage and tomato) were also established with reliable accuracy and precision under reverse-phase HPLC condition. Such results provided a baseline separation method for hexythiazox enantiomers under reverse-phase conditions and contributed to an environmental and health risk assessment of hexythiazox at enantiomer level.

## 1. Introduction

Hexythiazox((4RS,5RS)-5-(4-chlorophenyl)-N-cyclohexyl-4-methyl-2-oxothiazolidine-3-carboxamide, CAS: 78587-05-0, Figure 1) is a non-systemic acaricide widely used to control various mites on vegetables, fruits, cottons, pepper and flowers in agriculture and horticulture [1,2]. Although the specific mode of action (MoA) of hexythiazox is unknown, hexythiazox inhibits mite growth by a contact or stomach poison against eggs or early stages of mite development, thus the Insecticide Resistance Action Committee (IRAC) classify hexythiazox as mite growth inhibitors [3]. Commercial hexythiazox is a chiral pesticide and consists of two enantiomers, (4S, 5S)-hexythiazox and (4R, 5R)-hexythiazox, at a ratio of 1:1 [4]. Generally, the enantiomers of chiral pesticides have similar physical and chemical properties in non-chiral environments, whereas their biological behaviors may be completely different because of their different interaction capabilities with biomolecules in biological processes [5,6,7,8,9]. Moreover, the enantioselective accumulation, metabolism, degradation and bioactivity of chiral pesticide enantiomers have received great attention in recent years [10,11,12,13,14]. Thus, it is important and urgent to study the different biological behaviors of chiral pesticides at enantiomer level.

The separation of chiral pesticide enantiomers is a vital and fundamental step in enantiomer-specific risk assessment. Capillary electrophoresis (CE) [15], gas chromatography (GC) [16], normal-phase high-performance liquid chromatography (NP-HPLC) [17], reverse-phase high-performance liquid chromatography (RP-HPLC) [18], supercritical fluid chromatography (SFC) [19], ultra-performance convergence chromatography (UPCC) [20], cyclodextrin-modified micellar electrokinetic chromatography (CD-MEKC) [21], etc. were widely used for enantiomers separation. To date, high-performance liquid chromatography combined with different chiral stationary phases (CSPs) were considered as the most common and effective approach for chiral separation. Among the reported chiral stationary phases, polysaccharide-based CSPs including phenylcarbamates or benzoates derivatives, were the most commonly used CSPs due to their excellent capabilities to recognize enantiomers of chiral compounds, such as cellulose-tris-(3,5-dimethylphenylcarbamate), cellulose-tris-(3-chloro-4-methylphenylcarbamate), cellulose-tris-(4-methylbenzoate), cellulose-tris-(4-chloro-3-methylphenylcarbamate), amylose- tris-(3,5-dimethylphenylcarbamate), cellulose-tris-(3,5-dichlophenylcarbamate), etc. Generally, normal-phase HPLC is more suitable for chiral separation than reverse-phase HPLC due to better separation capability [17]. However, the reverse-phase HPLC has become a preferred and promising approach for enantiomers separation in recent years because of better solubility for polar compounds, lower background signal intensity, easier sample preparation procedures and excellent resolution under specific conditions [22]. Moreover, with the high development of mass spectrometry systems, reverse-phase HPLC is much more compatible with electrospray ionization (ESI) sources in mass spectrometry systems than normal-phase HPLC. Previous studies reported that reverse-phase HPLC coupled with tandem mass spectrometry was successfully applied in chiral pesticides separation including hexaconazole, epoxiconazole, metalaxyl, benalaxyl, myclobutanil, fenpropathrin, novaluron and permethrin, etc. [23,24,25,26,27,28]. To our best knowledge, there are only two studies that reported the chiral separation of hexythiazox enantiomers with normal-phase and reverse-phase HPLC. Wang et al. [29]. investigated the chiral separation of hexythiazox enantiomers with amylose-tris-(3,5-dimethylphenylcarbamate) chiral stationary phase under normal-phase HPLC condition and found the best resolution was R_s_ = 1.75 with an n-hexane/isopropanol ratio of 99.5/0.5. As for reverse-phase HPLC, Tian et al. [18] studied the chiral separation of hexythiazox enantiomers on cellulose-tris-(3,5-dimethylphenylcarbamate) and amylase-tris-(3,5-dimethylphenylcarbamate) based two chiral columns and found the best resolution was R_s_ = 0.96 on amylase-tris-(3,5-dimethylphenylcarbamate) based chiral column with acetonitrile/water ratio of 60/40. However, the baseline separation of hexythiazox enantiomers (R_s_ > 1.5) has not been achieved under reverse-phase conditions up to the present.

In the present study, the direct enantiomeric separation of hexythiazox enantiomers on Lux cellulose-1, Lux cellulose-2, Lux cellulose-3, Lux cellulose-4, Lux amylose-1 and Chirapak IC chiral columns were carefully investigated under reverse-phase HPLC condition. The effects of chiral stationary phase, temperature, thermodynamic parameters, the mobile phase component and mobile phase ratio on hexythiazox enantiomers separation were fully evaluated. Hexythiazox enantiomers received a baseline separation on the Lux cellulose-3 column with a maximum resolution of R_s_ = 2.09 (methanol/water) and R_s_ = 2.74 (acetonitrile/water), respectively. Furthermore, the residue analytical method for hexythiazox enantiomers in the environment (soil and water) and vegetable (cucumber, cabbage and tomato) were also established with reliable accuracy and precision. Such results provided a baseline separation method for hexythiazox enantiomers under reverse-phase HPLC condition and contributed to environmental and health risk assessment of hexythiazox at enantiomer level.

## 2. Materials and Methods

### 2.1. Chemicals and Reagents

Hexythiazox (purity = 98.0%) was purchased from Sigma-Aldrich (St. Louis, MO, USA). The stock solution of hexythiazox was prepared with methanol at 1000 mg∙L^−1^ and diluted to appropriate concentration. Acetonitrile (ACN) and methanol (MeOH) were HPLC grade and bought from Honeywell (Morristown, NJ, USA). Sodium chloride and anhydrous sodium sulfate were purchased from J&K Scientific Co., Ltd. (Beijing, China). Water (H_2_O) was purified with a Milli-Q system from Millipore (Billerica, MA, USA).

### 2.2. Apparatus and Chiral HPLC Analysis

Liquid chromatography was performed on an Agilent 1260 series HPLC system from Agilent Technologies (Palo Alto, CA, USA), which was equipped with a G1322A degasser, G1311B quatpump, G1316A column compartment, G1315D diode array detector (DAD) and G1329B autosampler with a 100 μL sample loop. The signals were collected and processed using an Agilent Chemstation. Hexythiazox enantiomers were separated on six chiral columns under reverse-phase HPLC condition, including Lux Cellulose-1 (cellulose-tris-(3,5-dimethylphenylcarbamate), 250 mm × 4.6 mm (internal diameter, i.d.), 5 μm), Lux Cellulose-2 (cellulose-tris-(3-chloro-4-methylphenylcarbamate), 250 mm × 4.6 mm (i.d.), 5 μm), Lux Cellulose-3 (cellulose-tris-(4-methylbenzoate), 250 mm × 4.6 mm (i.d.), 5 μm), Lux Cellulose-4 (cellulose-tris-(4-chloro-3-methylphenylcarbamate), 250 mm × 4.6 mm (i.d.), 5 μm), Lux Amylose-1 (amylose-tris-(3,5-dimethylphenylcarbamate), 250 mm × 4.6 mm (i.d.), 5 μm) and Chiralpak IC (cellulose-tris-(3,5-dichlophenylcarbamate), 250 mm × 4.6 mm (i.d.), 5 μm). The mobile phases were using solvent A (methanol or acetonitrile) and solvent B (water) with isocratic elution. In each run, the injection volume was 10 μL and the flow rate was 0.8 mL∙min^−1^ with the detection wavelength at 230 nm.

### 2.3. Method Validation

The performances of the analytical method were determined by linearity, precision, accuracy, stability, the limit of detection (LOD) and limit of quantitation (LOQ). The linear calibration curves of the method were the linear regression of the hexythiazox enantiomer area versus the injected concentration. Accuracy and precision were evaluated by the recovery and relative standard deviation (RSD) at three added levels (0.05, 0.5, 5 mg·kg^−1^) with five replicates, respectively. The LOD was regarded as the concentration of hexythiazox enantiomer that produced a signal-to-noise (S/N) ratio of 3, while the LOQ was defined as the lowest spiked concentration with acceptable RSD. The stability of the hexythiazox stock solution was checked monthly by injection of newly prepared working solution and found that hexythiazox was stable at −20 °C storage condition for at least 3 months.

### 2.4. Sample Preparation

Portions of 5.0 g homogenized samples (tomato, cucumber, cabbage, soil and water) were weighed to 50 mL polypropylene centrifuge tubes. Twenty-five milliliters acetonitrile and 5 mL ultrapure water were added to the tube. The mixture was placed in a THZ-D constant temperature oscillator for 20 min at 25 °C with 280 rpm rotational speed (Peiying, Jiangsu, China). After oscillation, the samples were exposed to ultrasonic vibration for 10 min, 2 g sodium chloride was added to the tube and shaken violently for 30 s and then centrifuged at 3500 rpm for 5 min. The upper acetonitrile layer was filtered through 10 g of anhydrous sodium sulfate for dehydration and the extraction steps were repeated with another 25 mL of acetonitrile. The combined extracts were evaporated to near dryness at 35 °C using a rotary vacuum evaporator and reconstituted in 1 mL methanol for HPLC analysis.

### 2.5. Data Analysis

Separation performances were evaluated by the following parameters: capacity factor (k = (t − t_0_)/t_0_)), separation factor (α = k_2_/k_1_) and resolution factor (R_s_ = (2(t_2_ − t_1_)/(w_1_ + w_2_))), where t is the retention time of hexythiazox enantiomers, t_0_ is the void time, k_1_ and k_2_ are capacity factors of the first and second eluted enantiomers of hexythiazox, w_1_ and w_2_ were peak width of the hexythiazox enantiomer.

Based on the capacity factor (k) and separation factor (α) obtained at different temperatures, the van’t Hoff equation was used to calculate the enthalpy (ΔH) and entropy (ΔS), which were key parameters to reveal the thermodynamic mechanism on hexythiazox enantiomers separation.

(1) $$\ln k=-\frac{\Delta H}{RT}+\frac{\Delta S}{R}+\ln\varphi$$

(2) $$\ln\alpha =-\frac{\Delta \Delta H}{RT}+\frac{\Delta \Delta S}{R}$$

where ∆H and ∆S were standard enthalpy and entropy between the chiral stationary phase and mobile phase, ∆∆H and ∆∆S were values of ∆H_2_ − ∆H_1_ and ∆S_2_ − ∆S_1_; where ∆H_1_, ∆H_2_, ∆S_1_ and ∆S_2_ represented the standard enthalpy and entropy values of the first and second eluted hexythiazox enantiomers, respectively, and φ was the column phase ratio. T was absolute temperature, k and R were the retention factors and universal gas constant (8.3144 J·(mol·K)^−1^), respectively. −∆H/R and (∆S/R + lnφ) were the slope and intercept of the linear regression-based Equation (1). Likewise, −∆∆H/R and ∆∆S/R could be obtained from the linear regression of lnα to 1/T (Equation (2)), respectively.

## 3. Results

### 3.1. Chiral Separation of Hexythiazox Enantiomers

**Figure 2:**
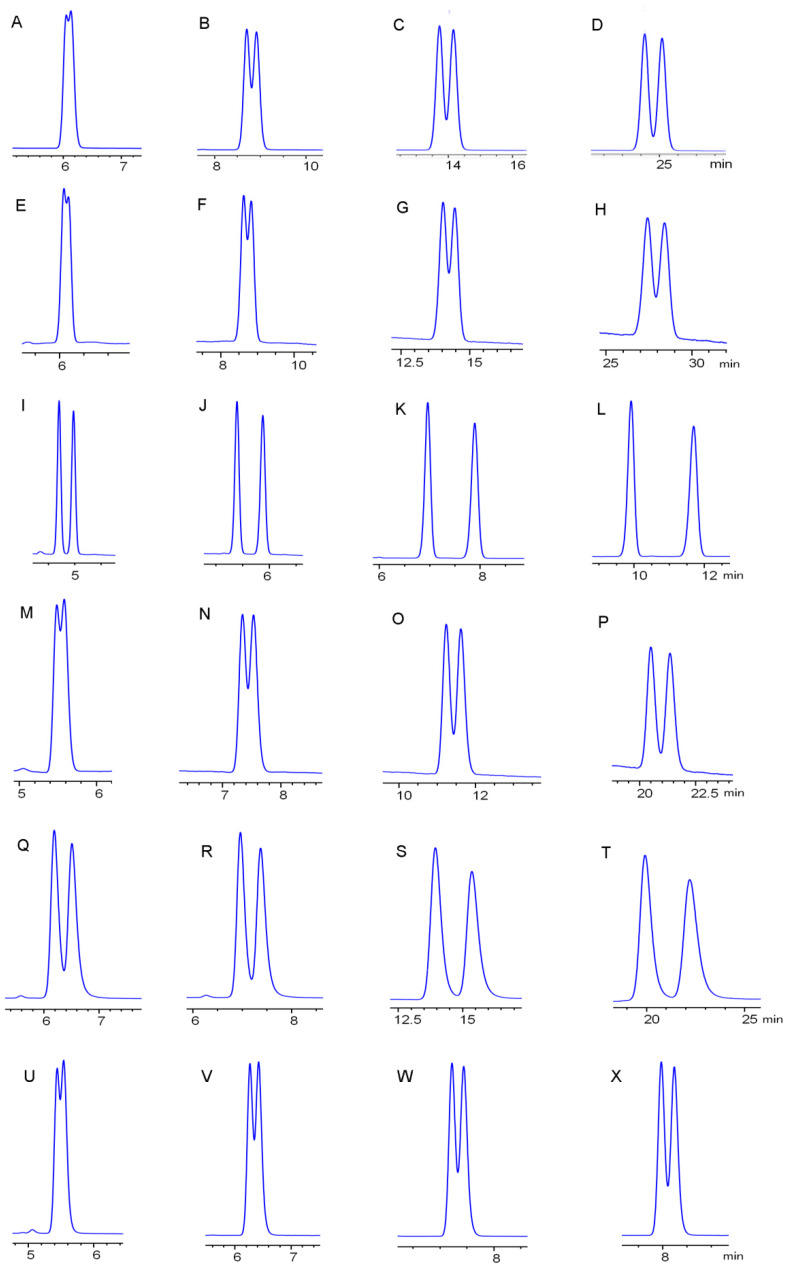
Chiral resolution chromatograms of hexythiazox enantiomers on Lux cellulose-1 (**A**–**D**), Lux cellulose-2 (**E**–**H**), Lux cellulose-3 (**I**–**L**), Lux cellulose-4 (**M**–**P**), Lux amylose-1 (**Q**–**T**) and Chirapak IC (**U**–**X**) columns at 20 °C with an ACN/H_2_O ratio of 90/10 (A,E,I,M,Q,U), 80/20 (B,F,J,N,R,V), 70/30 (C,G,K,O,S,W) and 60/40 (D,H,L,P,T,X), respectively.

The enantiomeric separation of hexythiazox enantiomers on six chiral columns was performed using methanol/water or acetonitrile/water as mobile phase at a flow rate of 0.8 mL·min^−1^ and 20 °C (Figure 2). Table S1 in supplementary materials summarizes the chiral resolution results, which include the capacity factors (k_1_, k_2_), separation factor (α) and resolution factor (R_s_). R_s_ > 1.50 is regarded as baseline separation. When methanol/water was used as mobile phase, hexythiazox enantiomers could be separated on the Lux cellulose-1, Lux cellulose-2, Lux cellulose-3 and Lux cellulose-4 columns, while Lux amylose-1 and Chirapak IC had no separation ability for hexythiazox enantiomers. Moreover, partial separation of hexythiazox enantiomers were obtained on Lux cellulose-1, Lux cellulose-2 and Lux cellulose-4 columns with a maximum R_s_ of 0.93, 0.81 and 0.73, respectively. Hexythiazox enantiomers could be completely separated on Lux cellulose-3 with maximum R_s_ = 2.09 at a methanol/water ratio of 100/0. When acetonitrile/water was used as mobile phase, hexythiazox enantiomers could be partially separated on Lux cellulose-1, Lux cellulose-2, Lux cellulose-4, Lux amylose-1 and Chirapak IC columns and completely separated on the Lux cellulose-3 column with a maximum R_s_ of 2.74 at acetonitrile/water ratio of 80/20. Different separation abilities were observed on the same chiral column between methanol/water and acetonitrile/water. Methanol was a polar protic solvent, which was a hydrogen-bond donor and acceptor, whereas acetonitrile was a polar aprotic solvent, which was just a weak hydrogen-bond acceptor. Thus, methanol/water and acetonitrile/water presented different separation abilities may be induced by different hydrogen-bond interactions involved in hexythiazox enantiomers, mobile phase and chiral stationary phase. Hexythiazox enantiomers received the best resolution on the Lux cellulose-3 column, which implied that the 4-methylbenzoate in Lux cellulose-3 column had better selectivity for hexythiazox enantiomers than other columns. In general, the lower ratio of organic solvent in mobile phase often leads to longer eluted time and higher separation resolution under reverse-phase HPLC conditions. In accordance with this phenomenon, the capacity factor (k) and resolution factor (R_s_) increased with a decreasing ratio of acetonitrile and methanol in mobile phase. However, the resolution factor (R_s_) on Lux cellulose-3 increased with increasing contents of methanol in mobile phase. Tian et al. [18] obtained partial separation of hexythiazox enantiomers on cellulose-tris-(3,5-dimethylphenylcarbamate) based chiral column with maximum R_s_ = 0.78 under reverse-phase condition. In the present study, hexythiazox enantiomers were completely separated on the Lux cellulose-3 column with maximum R_s_ = 2.09 and R_s_ = 2.74 using methanol/water and acetonitrile/water as mobile phase, respectively. Thus, our study provided a baseline separation method for hexythiazox enantiomers under reverse-phase HPLC for the first time.

### 3.2. Effects of Temperature on Hexythiazox Enantiomers Separation

Temperature is a vital factor for chiral separation and contributes to revealing the mechanism of chiral recognition. In the present study, the effects of column temperature on hexythiazox enantiomer separation were carefully investigated from 10 °C to 40 °C on Lux cellulose-1, Lux cellulose-2, Lux cellulose-3, Lux cellulose-4, Lux amylose-1 and Chirapak IC columns. Table 1 lists the chromatographic conditions and separation resolutions change with temperature. When methanol/water was used as mobile phase, hexythiazox enantiomers were separated on Lux cellulose-1 (85/15), Lux cellulose-2 (90/10), Lux cellulose-3 (90/10) and Lux cellulose-4 (90/10) in consideration of separation resolution and retention time. While acetonitrile/water was used as mobile phase, the two enantiomers were separated on Lux cellulose-1 (60/40), Lux cellulose-2 (70/30), Lux cellulose-3 (60/40) Lux cellulose-4 (70/30) Lux amylose-1 (60/40) and Chirapak IC (60/40). The results indicated that temperature had significant effects on hexythiazox enantiomer separation with different chiral stationary phases. Lower temperature generally results in longer retention time, higher resolution and wider peak (Figure S1 in supplementary materials). In accordance with this phenomenon, the capacity factor (k_1_, k_2_) and resolution factor (R_s_) decreased with the increasing trend of temperature on six chiral columns, no matter if methanol/water or acetonitrile/water were used as mobile phase. For example, the k_1_, k_2_ and R_s_ decreased from 1.77 to 1.16, 2.26 to 1.43 and 1.94 to 1.61, respectively on the Lux cellulose-3 column with methanol/water ratio of 90/10. Likewise, the k_1_, k_2_ and R_s_ values decreased from 1.31 to 1.00, 1.76 to 1.26 and 2.49 to 2.00, respectively, on the Lux cellulose-3 column with acetonitrile/water ratio of 60/40. However, temperature sometimes has little effect on chiral resolution. Zhang et al. [30] reported the chiral separation of lambda-cyhalothrin enantiomers on a Lux cellulose-3 column with a methanol/water ratio of 95/5 and found the best chiral resolution was obtained at 40 °C with a maximum R_s_ of 4.72.

### 3.3. Thermodynamic Parameters on Hexythiazox Enantiomers Separation

**Figure 3:**
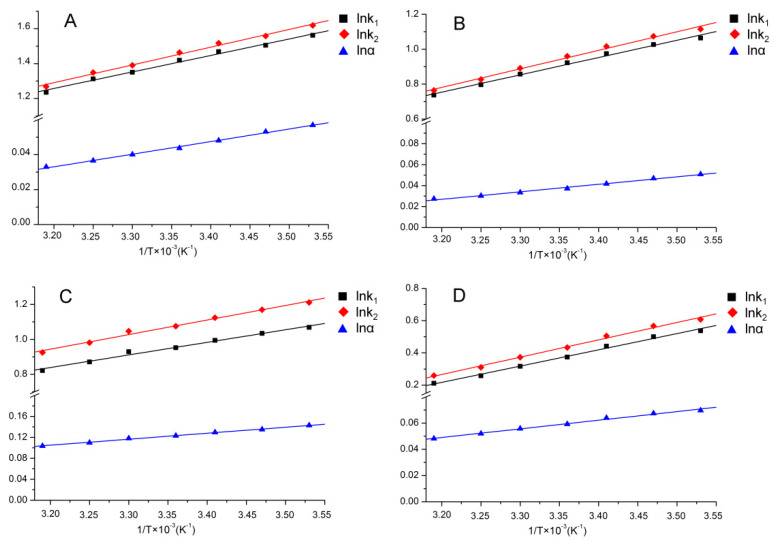
Van’t Hoff plots of hexythiazox on (**A**) Lux Cellulose-1 (acetonitrile/water, 60/40), (**B**) Lux Cellulose-2 (acetonitrile/water, 70/30), (**C**) Chirapak IC (acetonitrile/water, 60/40) and (**D**) Lux amylose-1 (acetonitrile/water, 60/40).

In order to determine the thermodynamic driving forces involved in hexythiazox enantiomers separation, the van’t Hoff equation was adopted to calculate the enthalpy (ΔH) and entropy (ΔS) values based on the capacity factor (k_1_, k_2_) and separation factor (α) obtained from Lux cellulose-1, Lux cellulose-2, Lux cellulose-3, Lux cellulose-4, Lux amylose-1 and Chirapak IC columns under different temperatures (Figure 3). The ΔH values of hexythiazox enantiomers on six chiral columns ranged from −5.95 to −14.08 KJ∙mol^−1^ when methanol/water and acetonitrile/water were used as mobile phase (Table 2). The negative values of ΔH implied that the processes of transfer hexythiazox enantiomers from mobile phase to chiral stationary phase were mainly driven by enthalpy. ∆∆H and ∆∆S were ranged from −0.54 to −1.60 KJ·mol^−1^ and −0.91 to −3.18 J·mol^−1^ on six chiral columns, respectively. The negative values of ∆∆H implied the ∆H values of the second enantiomer was more negative than the first eluted enantiomers, which indicated the interactions between the second eluted enantiomer and chiral stationary phase was stronger than the first eluted enantiomer. The negative values of ∆∆H also implied the lower temperature resulted in better chiral resolution, which was observed in temperature study. Studies reported that the main forces involved in enantiomeric separation were hydrogen bonding, π–π and dipole–dipole interaction. The good linearity of lnα versus 1/T implied that only one of these forces was involved in hexythiazox separation. Similarly, poor linearity of lnα versus 1/T would generally indicate multiple interaction forces existed in enantiomers separation [17,29,30].

### 3.4. Hexythiazox Enantiomers Analysis in Vegetable and Environment

**Figure 4:**
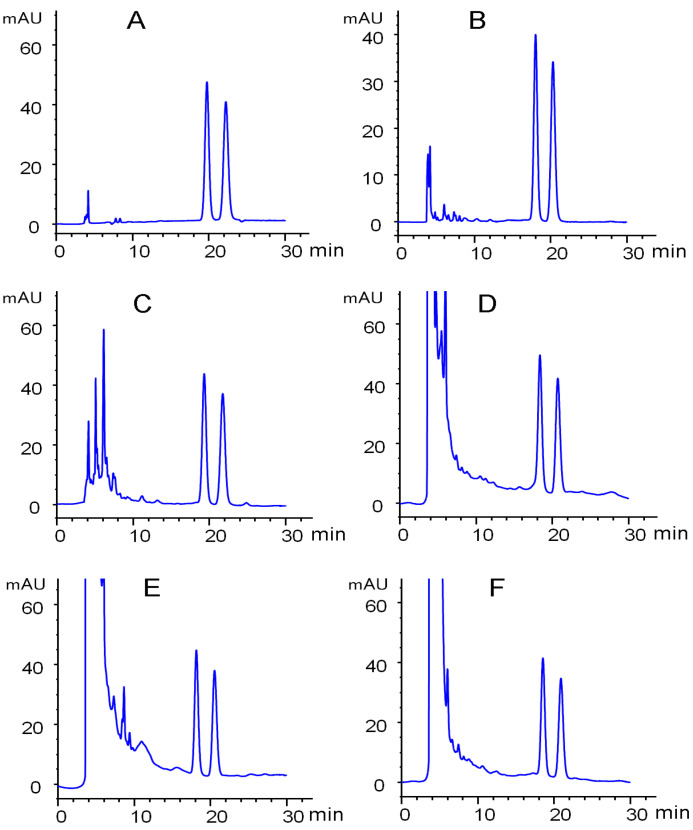
Representative chromatograms of hexythiazox enantiomers on the Lux Cellulose-3 column. (**A**) Standard solution; (**B**) extracted from water; (**C**) extracted from soil; (**D**) extracted from cucumber; (**E**) extracted from cabbage; (**F**) extracted from tomato.

According to baseline separation of hexythiazox enantiomers on the Lux cellulose-3 column, the quantitative analysis of hexythiazox enantiomers was validated in the environment (soil and water) and vegetable (cucumber, tomato and cabbage, Figure 4). Good linearities for the first eluted enantiomer (y = 34.324x + 7.5844, R^2^ = 0.9997) and second eluted enantiomer (y = 34.098x + 5.0445, R^2^ = 0.9998) were obtained from the concentration range of 0.05 to 10 mg·L^−1^. The accuracy and precision of the analytical method were determined by recovery and relative standard deviation (RSD) at three spiked levels in different matrices with five replicates. The reproducibility was evaluated by the interday precision, which represented the injection of the same sample on three sequential days. Table 3 listed the recovery and precision data of the developed analytical method. The recovery ranged from 86.54% to 99.84% and intraday precision ranged from 1.66% to 6.91% for the two hexythiazox enantiomers in cucumber, tomato, cabbage, soil and water samples. The LOD for hexythiazox enantiomers was 0.2 ng and the corresponding LOQ was 0.05 mg·kg^−1^ based on the lowest spiked concentration with an acceptable RSD. Such results indicated the developed method was reliable and effective for the residue analysis of hexythiazox enantiomers in vegetables and the environment.

## 4. Conclusions

In the context of this study, the direct enantiomeric separations of hexythiazox enantiomers on six chiral columns were comprehensively investigated by reverse-phase high-performance liquid chromatography. The effects of chiral stationary phase, temperature, thermodynamic parameters, mobile phase component and mobile phase ratio on hexythiazox enantiomers separation were carefully evaluated. Hexythiazox enantiomers received a baseline separation on the Lux cellulose-3 column with maximum R_s_ = 2.09 (methanol/water, 100/0) and R_s_ = 2.74 (acetonitrile/water, 50/50), respectively. Partial separations were achieved on the other five columns where the maximum R_s_ ranged from 0.35 to 0.93. Furthermore, Lux amylose-1 and Chirapak IC had no separation ability for hexythiazox enantiomer when methanol/water was used as mobile phase. A temperature study indicated that capacity factor (k) and resolution factor (R_s_) decreased with column temperature increasing from 10 °C to 40 °C. The enthalpy (ΔH) and entropy (ΔS) involved in hexythiazox separation were also calculated and demonstrated the lower temperature contributed to better separation resolution. Moreover, the residue analytical method for hexythiazox in the environment (soil and water) and vegetable (cucumber, cabbage and tomato) were also established with reliable accuracy and precision under reverse-phase HPLC conditions. Such results provided a baseline separation method for hexythiazox enantiomers under reverse-phase conditions and contributed to environmental and health risk assessment of hexythiazox at enantiomer level.

## Figures and Tables

**Figure 1:**
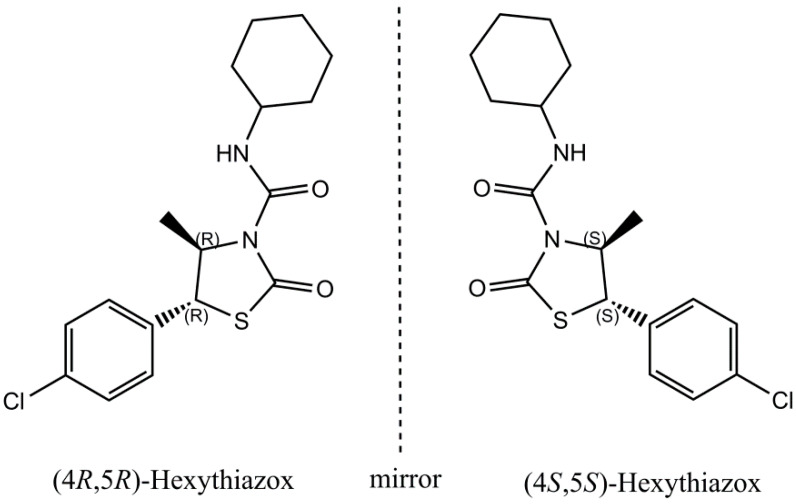
Chemical structure of hexythiazox enantiomers.

**Table 1:** Effects of temperature on hexythiazox separation with six chiral columns.

Stationary Phase	Mobile Phase (*v*/*v*)	Temperature (°C)	k_1_	k_2_	α	Rs	Mobile Phase (*v*/*v*)	Temperature (°C)	k_1_	k_2_	α	Rs
Lux Cellulose-1	MeOH/H_2_O 85/15	10	3.83	4.18	1.09	0.79	ACN/H_2_O 60/40	10	4.77	5.05	1.06	0.73
		15	3.59	3.89	1.08	0.70		15	4.50	4.75	1.05	0.67
		20	3.25	3.50	1.08	0.68		20	4.34	4.56	1.05	0.66
		25	3.00	3.20	1.07	0.66		25	4.13	4.32	1.04	0.62
		30	2.64	2.80	1.06	0.65		30	3.86	4.02	1.04	0.59
		35	2.49	2.63	1.06	0.59		35	3.72	3.85	1.04	0.54
		40	2.30	2.39	1.04	0.44		40	3.44	3.56	1.03	0.48
Lux Cellulose-2	MeOH/H_2_O 90/10	10	2.12	2.28	1.08	0.88	ACN/H_2_O 70/30	10	2.90	3.05	1.05	0.72
		15	2.09	2.24	1.07	0.76		15	2.79	2.93	1.05	0.69
		20	1.93	2.05	1.06	0.67		20	2.65	2.76	1.04	0.63
		25	1.78	1.88	1.06	0.63		25	2.51	2.61	1.04	0.59
		30	1.64	1.73	1.05	0.60		30	2.36	2.44	1.03	0.52
		35	1.52	1.60	1.05	0.55		35	2.22	2.29	1.03	0.51
		40	1.41	1.47	1.04	0.52		40	2.09	2.15	1.03	0.48
Lux Cellulose-3	MeOH/H_2_O 90/10	10	1.77	2.26	1.28	1.94	ACN/H_2_O 60/40	10	1.31	1.76	1.35	2.49
		15	1.62	2.05	1.27	1.83		15	1.28	1.70	1.33	2.45
		20	1.57	1.98	1.26	1.79		20	1.23	1.62	1.32	2.28
		25	1.46	1.83	1.25	1.77		25	1.16	1.51	1.30	2.20
		30	1.37	1.70	1.25	1.70		30	1.11	1.43	1.29	2.15
		35	1.25	1.56	1.24	1.68		35	1.05	1.34	1.27	2.01
		40	1.16	1.43	1.23	1.61		40	1.00	1.26	1.26	2.00
Lux Cellulose-4	MeOH/H_2_O 90/10	10	1.60	1.70	1.06	0.66	ACN/H_2_O 70/30	10	2.18	2.32	1.06	0.76
		15	1.54	1.63	1.06	0.62		15	2.11	2.23	1.06	0.74
		20	1.43	1.51	1.05	0.57		20	2.04	2.15	1.05	0.73
		25	1.32	1.38	1.05	0.54		25	1.95	2.04	1.05	0.68
		30	1.23	1.29	1.04	0.50		30	1.86	1.94	1.04	0.66
		35	1.17	1.22	1.04	0.47		35	1.72	1.79	1.04	0.61
		40	1.08	1.11	1.03	0.42		40	1.62	1.67	1.03	0.53
Lux Amylose-1	-	10	-	-	-	-	ACN/H_2_O 60/40	10	2.91	3.36	1.15	0.82
		15	-	-	-	-		15	2.81	3.22	1.14	0.79
		20	-	-	-	-		20	2.70	3.08	1.14	0.76
		25	-	-	-	-		25	2.59	2.93	1.13	0.73
		30	-	-	-	-		30	2.53	2.85	1.13	0.70
		35	-	-	-	-		35	2.39	2.67	1.12	0.68
		40	-	-	-	-		40	2.27	2.52	1.11	0.65
Chiralpak IC	-	10	-	-	-	-	ACN/H_2_O 60/40	10	1.71	1.84	1.07	0.72
		15	-	-	-	-		15	1.65	1.76	1.07	0.69
		20	-	-	-	-		20	1.55	1.66	1.07	0.68
		25	-	-	-	-		25	1.45	1.54	1.06	0.65
		30	-	-	-	-		30	1.37	1.45	1.06	0.64
		35	-	-	-	-		35	1.29	1.36	1.05	0.62
		40	-	-	-	-		40	1.24	1.30	1.05	0.55

**Table 2:** Van’t Hoff equation and thermodynamic parameters of hexythiazox enantiomers on six chiral columns.

Column	Mobile Phase (*v*/*v*)	lnk = −△H/RT + △S/R + lnφ	R^2^	△H (KJ mol^-1^)	△S/R+ lnφ	lnα = −∆∆H/RT + ∆∆S/R	R^2^	△△H (KJ mol^-1^)	△△S (J mol^-1^)
Lux Cellulose-1	MeOH/H_2_O 85/15	lnk_1_ = 1560.1/T−4.1536	0.994	−12.97	−4.55	lnα = 132.9/T-0.3805	0.962	−1.10	−3.16
		lnk_2_ = 1693/T−4.5341	0.995	−14.08	−4.78				
	ACN/H_2_O 60/40	lnk_1_ = 936.25/T−1.7378	0.988	−7.78	−0.35	lnα = 71.652/T-0.1962	0.998	−0.60	−1.63
		lnk_2_ = 1007.9/T−1.934	0.990	−8.38	−0.42				
Lux Cellulose-2	MeOH/H_2_O 90/10	lnk_1_ = 1276.6/T−3.7204	0.980	−10.61	−3.56	lnα = 90.966/T-0.2477	0.996	−0.76	−2.06
		lnk_2_ = 1367.5/T−3.9682	0.982	−11.37	−5.55				
	ACN/H_2_O 70/30	lnk_1_ = 983.82/T−2.3939	0.990	−8.18	−3.71	lnα = 71.12/T-0.2006	0.995	−0.59	−1.67
		lnk_2_ = 1054.9/T−2.5946	0.992	−8.77	−5.99				
Lux Cellulose-3	MeOH/H_2_O 90/10	lnk_1_ = 1211/T−3.7009	0.986	−10.07	−5.37	lnα = 99.749/T-0.1097	0.994	−0.83	−0.91
		lnk_2_ = 1310.8/T−3.8105	0.988	−10.90	−5.78				
	ACN/H_2_O 60/40	lnk_1_ = 824.51/T−2.6256	0.986	−6.85	−1.43	lnα = 192.8/T-0.3828	0.997	−1.60	−3.18
		lnk_2_ = 1017.3/T−3.0084	0.989	−8.46	−1.63				
Lux Cellulose-4	MeOH/H_2_O 90/10	lnk_1_ = 1193.9/T−3.7285	0.992	−9.93	−6.93	lnα = 74.735/T-0.2033	0.988	−0.62	−1.69
		lnk_2_ = 1268.7/T−3.9318	0.992	−10.55	−9.69				
	ACN/H_2_O 70/30	lnk_1_ = 872.27/T−2.2803	0.967	−7.25	−1.05	lnα = 80.151/T-0.2215	0.997	−0.67	−1.84
		lnk_2_ = 952.42/T−2.5018	0.971	−7.92	−0.97				
Lux Amylose-1	ACN/H_2_O 60/40	lnk_1_ = 715.75/T−1.4515	0.987	−5.95	−1.43	lnα = 113.47/T-0.258	0.996	−0.94	−2.15
		lnk_2_ = 829.22/T−1.7095	0.989	−6.89	−1.63				
Chirapak IC	ACN/H_2_O 60/40	lnk_1_ = 1003.6/T−2.9947	0.995	−8.34	−1.05	lnα = 65.454/T-0.1604	0.992	−0.54	−1.33
		lnk_2_ = 1069.1/T-3.1551	0.995	−8.89	−0.97				

**Table 3:** Recovery and precision of the reverse-phase HPLC method for the measurement of hexythiazox enantiomers using the Lux Cellulose-3 column.

Compound	Matrix	Spiked Levels (mg∙kg^−1^ or mg∙L^−1^)	Intraday ^a^						Interday ^b^	
			Day 1		Day 2		Day 3			
			Recovery (%)	RSD ^c^ (%)	Recovery (%)	RSD (%)	Recovery (%)	RSD (%)	Recovery (%)	RSD (%)
E1	soil	0.05	89.38	4.87	88.00	5.31	87.21	5.59	88.19	5.36
		0.5	94.33	4.52	98.93	3.12	90.82	2.67	94.69	4.98
		5	96.58	3.48	97.55	2.80	93.34	2.62	95.83	3.54
	water	0.05	81.40	5.91	84.91	5.34	87.76	6.14	84.69	6.58
		0.5	88.77	4.38	92.99	3.92	94.36	5.01	92.04	5.16
		5	93.96	4.89	94.18	2.05	97.46	1.66	95.20	3.59
	cucumber	0.05	95.94	6.29	91.75	6.68	88.75	3.87	92.15	6.63
		0.5	93.31	5.13	92.49	3.63	95.79	5.33	93.86	5.00
		5	97.50	2.20	96.49	3.63	96.74	3.26	96.91	3.12
	tomato	0.05	89.65	5.21	90.54	4.01	89.30	3.80	89.83	4.42
		0.5	96.94	6.00	96.27	1.93	98.71	4.30	97.31	4.54
		5	97.36	3.78	96.56	2.94	97.51	1.98	97.14	3.02
	cabbage	0.05	90.36	4.76	89.39	4.22	91.65	3.85	90.47	4.41
		0.5	97.43	3.00	96.06	1.93	99.84	3.90	97.78	3.47
		5	98.79	3.77	97.71	3.19	98.88	2.01	98.46	3.13
E2	soil	0.05	91.38	5.45	88.84	6.38	89.09	5.54	89.77	5.94
		0.5	95.17	4.75	93.41	3.30	93.16	2.15	93.91	3.71
		5	97.29	3.60	99.12	3.42	95.72	1.69	97.38	3.36
	water	0.05	86.54	5.62	89.10	6.91	87.61	6.01	87.75	6.33
		0.5	91.21	2.31	96.71	3.49	99.44	5.54	95.79	5.44
		5	95.07	4.27	93.90	2.90	97.15	2.21	95.37	3.52
	cucumber	0.05	96.18	6.58	97.10	3.25	92.06	5.46	95.11	5.75
		0.5	94.55	2.67	94.64	1.65	93.86	4.02	94.35	2.96
		5	96.28	2.65	98.92	4.15	97.49	2.86	97.56	3.49
	tomato	0.05	89.57	3.08	89.16	6.44	91.84	2.06	90.19	4.45
		0.5	100.46	4.89	98.84	3.79	102.48	5.71	100.60	5.10
		5	97.25	3.32	97.07	3.52	97.36	2.88	97.23	3.25
	cabbage	0.05	94.86	2.93	94.50	4.05	92.78	3.98	94.05	3.81
		0.5	97.60	4.66	95.67	3.70	101.50	2.95	98.26	4.54
		5	101.39	3.12	96.39	4.22	99.27	1.47	99.02	3.75

^a^ Intraday RSD (*n* = 5). ^b^ Interday RSD (*n* = 15).^c^ RSD, relative standard deviation.

## Supplementary Materials

The following are available online at https://www.mdpi.com/1660-4601/17/10/3453/s1, Table S1: Enantiomeric separation results of hexythiazox enantiomers on six chiral columns at 20 °C using methanol/water or acetonitrile/water as mobile phase; Figure S1: The effects of temperature on hexythiazox enantiomers separation with Lux cellulose-3 column (methanol/water (90/10), A 10 °C, B 20 °C, C 30 °C, D 40 °C) and Lux cellulose-2 column (methanol / water (90/10), E 10°C, F 20 °C, G 30°C, H 40 °C).

## Abbreviations

CSP, chiral stationary phase; CD, circular dichroism; GC, gas chromatography; HPLC, high-performance liquid chromatography; LOD, limit of detection; RSD, relative standard deviation.
