# Supplementary material for: Direct Enantiomeric Separation and Determination of Hexythiazox Enantiomers in Environment and Vegetable by Reverse-Phase High-Performance Liquid Chromatography
Source: Int J Environ Res Public Health. 2020 May 15;17(10):3453. doi: 10.3390/ijerph17103453 (PMC7277754; doi:10.3390/ijerph17103453)
Supplement: Supplementary file 1 [file ijerph-17-03453-s001.pdf]

Supplementary Materials

Direct enantiomeric separation and determination of hexythiazox enantiomers in environment and vegetable by reverse-phase high performance liquid chromatography

Ping Zhang[1,2,3,*], Sheng Wang[1,2], Dongmei Shi[1,2], Yangyang Xu[1,2], Furong Yang[1,2], Xile Deng[4], Yuhan He[1,2] and Lin He [1,2,3,*]

[1] Key Laboratory of Entomology and Pest Control Engineering, College of Plant Protection, Southwest University, Chongqing, China; zpcauz@163.com (S.W.); zp8708@163.com (Y. X.);

[2] Academy of Agricultural Sciences, Southwest University, Chongqing, China; shidm48@163.com (D. S.); yfr200111@163.com (F. Y.); hm20161027@163.com (Y. H.).

[3] State Cultivation Base of Crop Stress Biology for Southern Mountainous Land of Southwest University, Southwest University, Chongqing, China; pingz@swu.edu.cn(P. Z.); helab01@ swu.edu.cn(L. H.)

[4] Key Laboratory for Biology and Control of Weeds, Biotechnology Research Institute, Hunan Academy of Agricultural Sciences, Changsha, China; dengxile@hunaas.cn(X.D.)

* Correspondence: pingz@swu.edu.cn; Tel.: +86-23-68251514(P. Z.); helab01@ swu.edu.cn; Tel.: +86-23-68254105(L. H.)

Page 2: Table S1 Enantiomeric separation results of hexythiazox enantiomers on six chiral columns at 20℃ using methanol/water or acetonitrile/water as mobile phase.

Page 3: Figure S1 The effects of temperature on hexythiazox enantiomers separation with Lux cellulose-3 column (methanol/water (90/10), A 10℃, B 20℃, C 30℃, D 40℃) and Lux cellulose-2 column (methanol / water (90/10), E 10℃, F 20℃, G 30℃, H 40℃).

Table S1 Enantiomeric separation results of hexythiazox enantiomers on six chiral columns at 20℃ using methanol/water or acetonitrile/water as mobile phase.

column	methanol/water					acetonitrile/water				
	Ratio(v/v)	k_1	k2	α	Rs	Ratio(v/v)	k1	k2	α	Rs
Lux Cellulose-1	95/5	1.17	1.27	1.09	0.73	90/10	0.60	0.63	1.03	0.35
	90/10	1.96	2.11	1.08	0.77	80/20	1.57	1.63	1.04	0.56
	85/15	3.18	3.43	1.08	0.82	70/30	3.17	3.30	1.04	0.66
	80/20	5.28	5.72	1.08	0.87	60/40	6.68	6.97	1.04	0.73
	75/25	9.13	9.94	1.09	0.93	50/50	10.43	10.96	1.05	0.87
Lux Cellulose-2	95/5	1.04	1.12	1.07	0.64	90/10	0.70	0.73	1.04	0.38
	90/10	2.13	2.27	1.07	0.71	80/20	1.31	1.36	1.04	0.54
	85/15	5.46	5.70	1.05	0.75	70/30	2.68	2.79	1.04	0.67
	80/20	7.64	8.07	1.06	0.81	60/40	5.83	6.07	1.04	0.77
Lux Cellulose-3	100/0	0.57	0.82	1.42	2.09	90/10	0.18	0.25	1.39	0.99
	95/5	0.99	1.32	1.33	1.89	80/20	0.33	0.44	1.36	1.61
	90/10	1.58	1.98	1.26	1.71	70/30	0.68	0.91	1.33	2.08
	85/15	2.68	3.20	1.20	1.44	60/40	1.36	1.78	1.31	2.29
	80/20	4.54	5.19	1.14	1.13	50/50	3.28	4.26	1.30	2.74
Lux Cellulose-4	90/10	1.39	1.46	1.05	0.56	90/10	0.48	0.51	1.05	0.45
	85/15	2.59	2.73	1.05	0.67	80/20	0.93	0.98	1.05	0.56
	80/20	5.03	5.27	1.05	0.69	70/30	2.06	2.16	1.05	0.73
	75/25	10.00	10.44	1.04	0.73	60/40	4.05	4.26	1.05	0.78
Lux Amylose-1	100/0	/	/	/	/	90/10	0.54	0.62	1.15	0.64
	95/5	/	/	/	/	80/20	0.72	0.82	1.14	0.65
	90/10	/	/	/	/	70/30	1.24	1.41	1.14	0.74
	85/15	/	/	/	/	60/40	2.31	2.65	1.15	0.86
	80/20	/	/	/	/	50/50	3.66	4.19	1.15	0.90
Chirapak IC	90/10	/	/	/	/	90/10	0.26	0.29	1.09	0.43
	85/15	/	/	/	/	80/20	0.44	0.47	1.08	0.61
	80/20	/	/	/	/	70/30	0.64	0.68	1.08	0.62
	75/25	/	/	/	/	60/40	0.79	0.85	1.08	0.67
	70/30	/	/	/	/	50/50	3.27	3.47	1.06	0.76

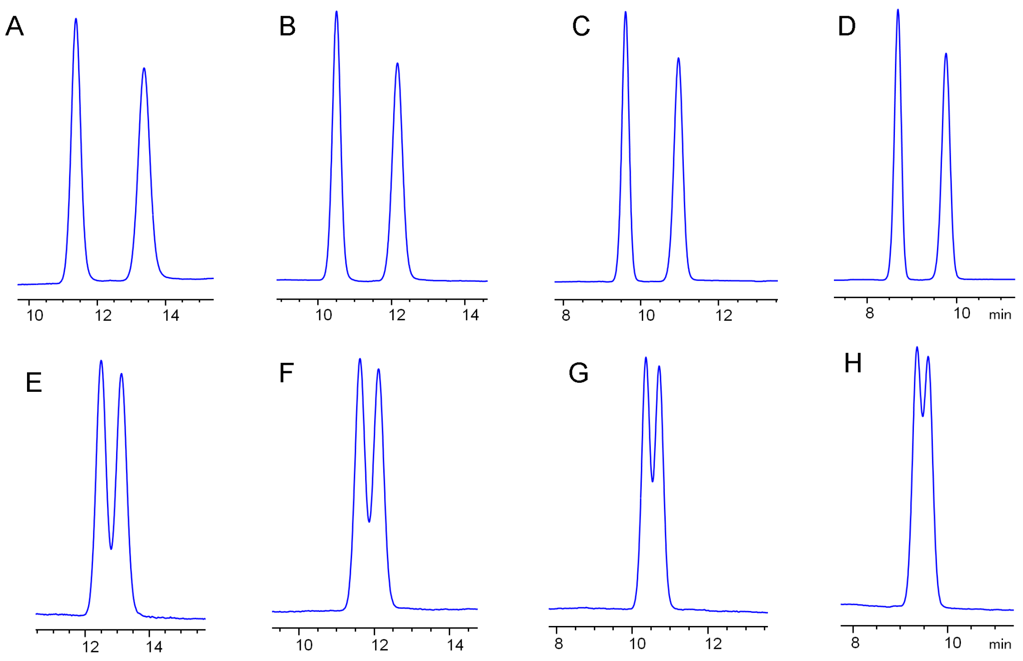

Figure S1 The effects of temperature on hexythiazox enantiomers separation with Lux cellulose-3 column (methanol/water (90/10), A 10℃, B 20℃, C 30℃, D 40℃) and Lux cellulose-2 column (methanol / water (90/10), E 10℃, F 20℃, G 30℃, H 40℃).
